# The Association of Ambient Temperature and Violent Crime

**DOI:** 10.1038/s41598-017-06720-z

**Published:** 2017-07-28

**Authors:** Jari Tiihonen, Pirjo Halonen, Laura Tiihonen, Hannu Kautiainen, Markus Storvik, James Callaway

**Affiliations:** 1 Department of Forensic Psychiatry, University of Eastern Finland, Niuvanniemi Hospital, FI-70240 Kuopio, Finland; 2 Department of Clinical Neuroscience, Karolinska Institutet, S-171 76 Stockholm, Sweden; 3 University of Jyväskylä, FI-40014 Jyväskylän yliopisto, Jyväskylä Finland; 4 University of Tampere, FI-33100 Tampere, Finland; 5 Unit of Primary Care, Helsinki University Central Hospital, and Department of General Practice, University of Helsinki, FI-00014 Helsingin yliopisto, Helsinki, Finland; 6 Department of Pharmacology and Toxicology, University of Eastern Finland, FI-70211 Kuopio, Finland; 7 Department of Pharmaceutical Chemistry, University of Eastern Finland, FI-70211 Kuopio, Finland

## Abstract

It is controversial if global warming will result into increased crime and conflict rate, and no causal neurobiological mechanisms have been proposed for the putative association between ambient temperature and aggressive behavior. This study shows that during 1996–2013, ambient temperature explained 10% of variance in the violent crime rate in Finland, corresponding to a 1.7% increase/degree centigrade. Ambient temperature also correlated with a one month delay in circannual changes in peripheral serotonin transporter density among both offenders and healthy control subjects, which itself correlated strongly with the monthly violent crime rate. This suggests that rise in temperature modulates serotonergic transmission which may increase impulsivity and general human activity level, resulting into increase in social interaction and risk of violent incidents. Together, these results suggest that the effect of ambient temperature on occurrence of violent crime is partly mediated through the serotonergic system, and that a 2 °C increase in average temperatures would increase violent crime rates by more than 3% in non-tropical and non-subtropical areas, if other contributing factors remained constant.

## Introduction

The effect of global warming on the occurrence of violence and conflict have received increasing attention during the last 10 years^1–4^. Some studies have suggested that climate has a substantial effect on conflicts and violence^1, 2^, while others have not found any significant association^5, 6^. The methodology and measurement of violence and related issues is complex, since a large number of factors are involved; e.g., high temperature, decreased rainfall, overpopulation, decreased food supplies, and other social, economical and political conditions. Instead of studying conflicts and wars, the present study investigated the relationship between ambient temperature and the occurrence of interpersonal violent behaviors. Several studies have reported a linear or curvilinear relationship between high temperatures and violent offences^7–10^. Several models of the temperature-aggression hypothesis have been suggested, including physiological-thermoregulatory model^11^. However, to our knowledge, no causal neurobiological mechanisms have been proposed thus far.

Several studies have shown that seasonal variation in the occurrence of suicide is associated with concomitant variations of meteorological and biochemical variables^12–15^. Thus far, concerning weather conditions, high correlations have been reported between suicide rates and ambient temperature, relative humidity, and the amount of sunlight^12^. Among biological factors, the serotonergic system has a major role in regulating violent behavior^13^. Ambient temperature, seasonality and relative humidity have been observed to be associated with B_max_ values from platelet [^3^H]-paroxetine and [^3^H]-citalopram binding studies^14, 15^. These findings imply that ambient environmental conditions modulate serotonin (5-HT) function, and that changes in 5-HT function lead to variations in human behavior. This is in line with previous studies which indicate that low CSF-5-hydroxy-indoleacetic acid (5-HIAA) levels are associated with violent suicide and impulsive violence directed towards other persons^16, 17^. However, to our knowledge, no associations have been reported between seasonal variations, violent offences and serotonergic biomarkers.

We previously reported a seasonal variation in the homicide occurrence in Finland and suggested that this variation is associated with circannual rhythms of 5-HT function^18^, and that the seasonal variation of peripheral serotonin transporter is more profound among violent offenders than healthy subjects^15^. Earlier studies have already demonstrated that platelet serotonin transporters (SERT) are peripheral biomarkers of serotonergic neurotransmission in the brain^19^. These results encouraged us to study the associations between the occurrence of violent crimes, weather conditions and platelet SERT density. Based on earlier studies, we hypothesized that there may a positive correlation between the amount of sunshine, ambient temperature and the occurrence of violent crime. A second hypothesis stated that there is a negative correlation between the density (B_max_) of platelet SERT (i.e., citalopram binding) and the occurrence of violent crime, as low levels of platelet paroxetine binding^20–24^ (i.e., SERT density) and low 5-HT re-uptake site densities in the midbrain^25^ are associated with aggressive behavior.

## Results

**Figure 1:**
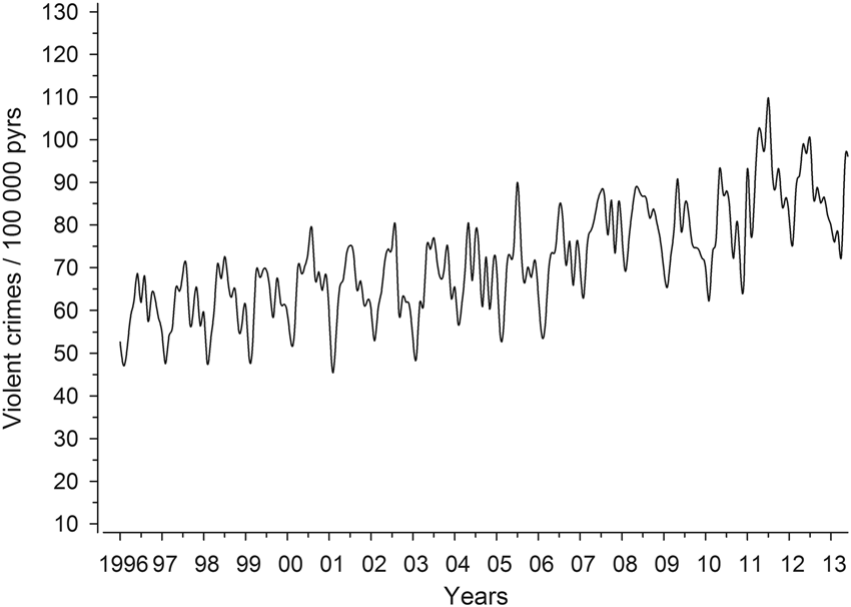
The incidence of violent crime per 100,000 person years in Finland between January 1996 and December 2013.

**Figure 2:**
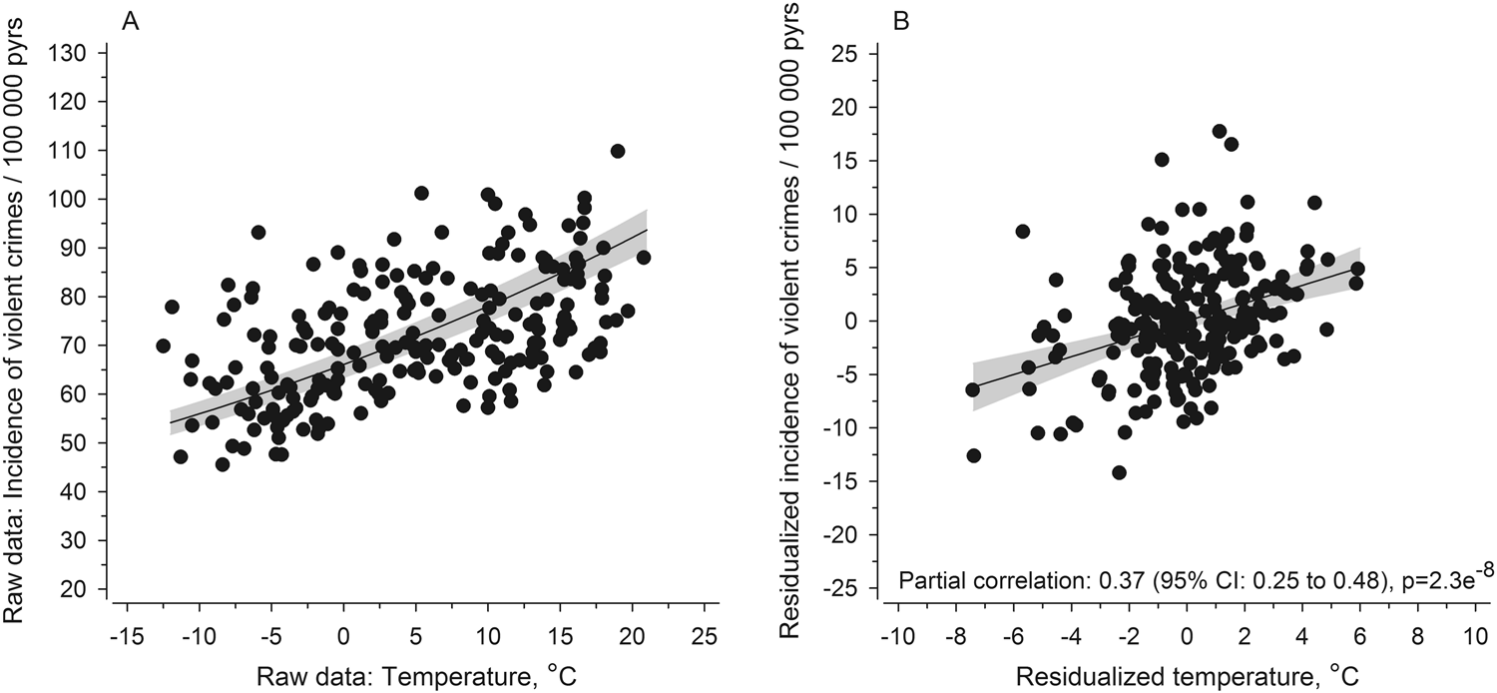
Panel A: The correlation between the monthly ambient temperature and monthly violent crime rate per 100,000 person years in Finland between January 1996 and December 2013 (r = 0.51, N = 216, p = 7.6 × 10^−16^). Panel B: Residualized (controlled for seasonality and time trends) relationship between violent crime and temperature values.

Figure 1 shows the incidence of violent crime during 1996–2013 in Finland, and Fig. 2 shows the correlation between mean monthly ambient temperature and the number of violent crimes. As expected, a strong correlation was observed between the monthly violent crime rate and monthly mean ambient temperature (r = 0.51, N = 216, p = 7.6 × 10^−16^). The corresponding correlation between the monthly sum of direct sunshine hours (normalized with the number of days/month) vs. monthly violent crime was substantially weaker (r = 0.24, N = 216, p = 1.9 × 10^−4^) than for ambient temperature vs. monthly violent crime rate (p = 1.4^−07^ for the comparative difference). The correlation co-efficient between monthly violent crime rate and mean ambient temperature of the preceding month (i.e., one month earlier) was 0.40 (N = 216, p = 1.8 × 10^−9^).

**Figure 3:**
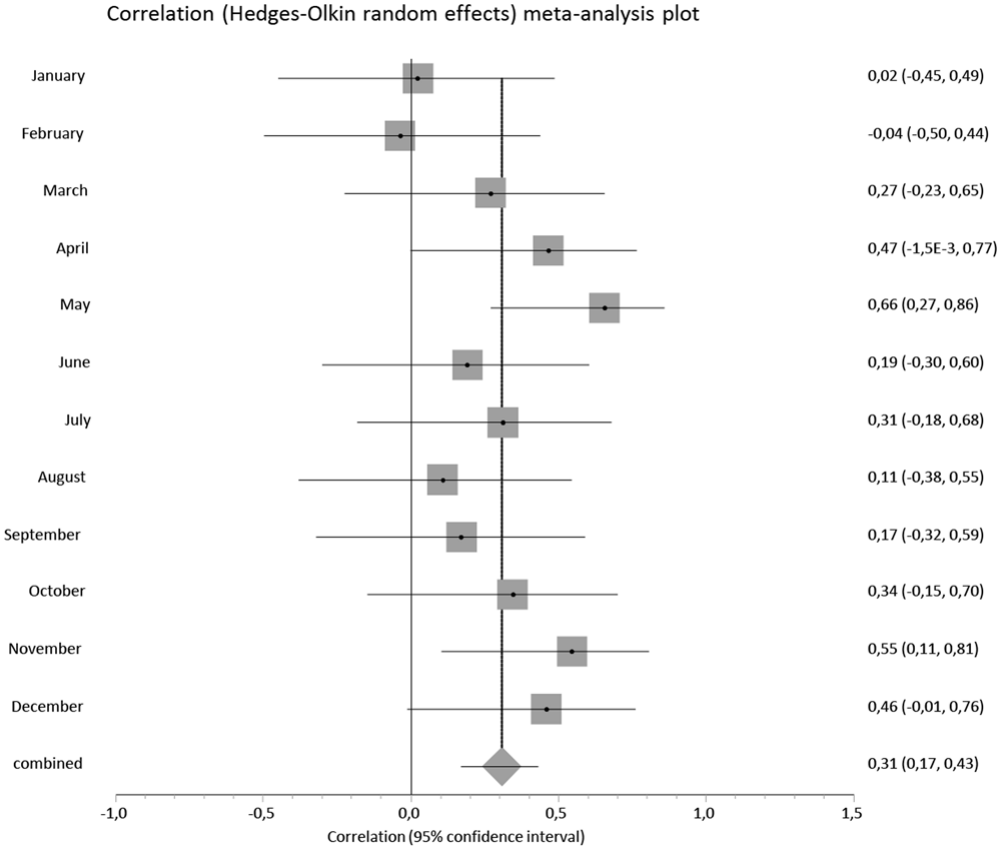
The correlation between violent crime rate and monthly mean ambient temperature in Finland, stratified by calendar month. During the 18-year follow-up, the pooled correlation co-efficient was 0.31 (N = 18, z-score 4.27, p = 2.0 × 10^−5^).

**Figure 4:**
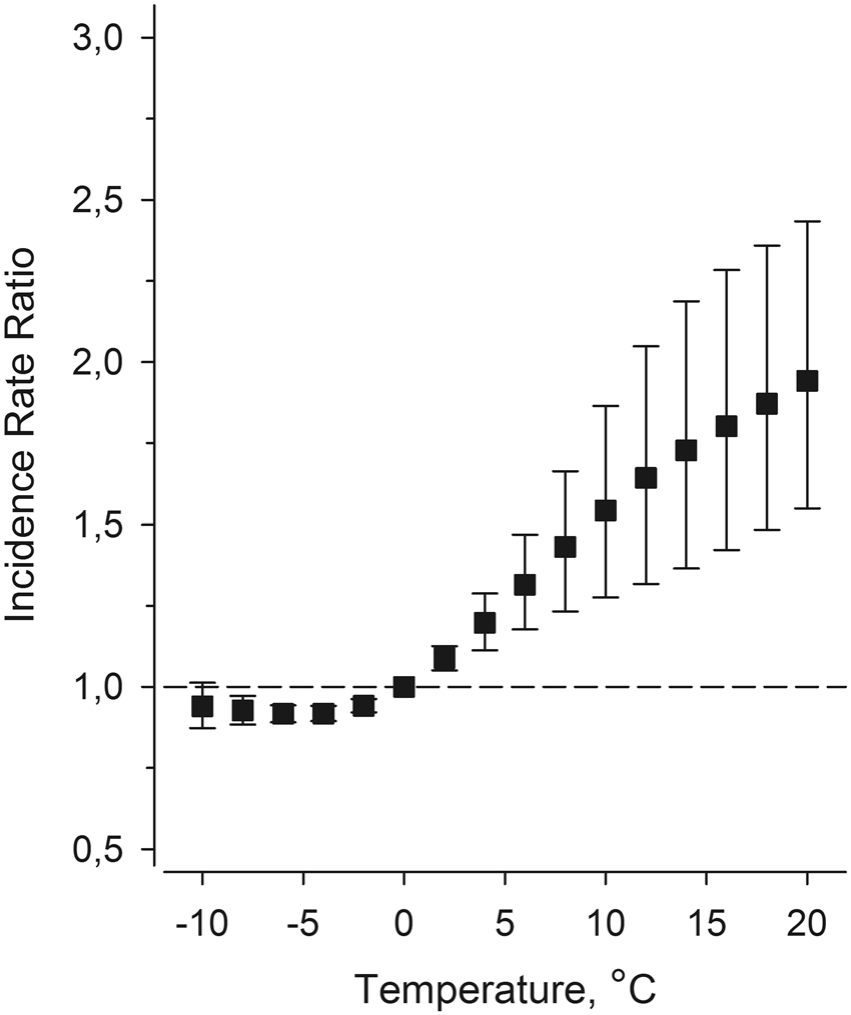
Incidence rate ratios of violent crime rates as a function of ambient temperature in Finland. Data were fitted by using a Poisson regression model with restricted cubic splines of 5 knots. The reference value of temperature is 0 °C. The increase per degree centigrade is 1.7% (95% CI 1.1 to 2.4).

Figure 3 shows a correlation between ambient temperature vs. violent crime rate stratified by month. Overall, ambient temperature explained 10% of the variance in violent crime rates (r = 0.31, 95%CI 0.17–0.43; p = 2.0 × 10^−5^). The correlation between sunshine hours and violent crime became weakly negative when months were stratified (r = −0.19, 95%CI −0.05 to −0.33). (Supplementary Figure S1). The IRR is shown in Fig. 4 as a function of temperature, with a 1.7% increase in violent crime incidence per degree centigrade.

**Figure 5:**
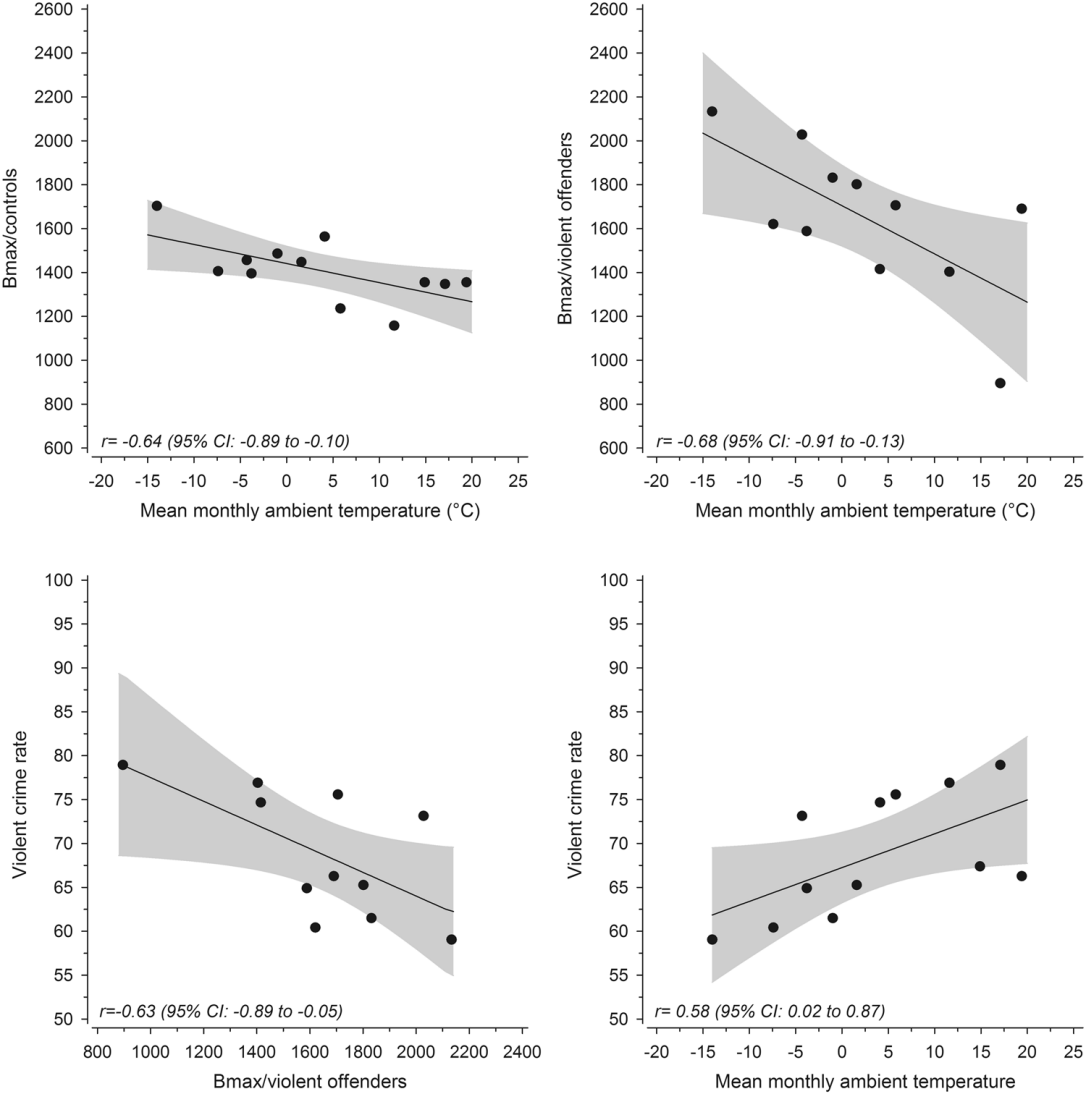
The correlations between ambient temperature of preceding month, peripheral SERT denties (B_max_, as fM of ligand/mg of protein) and violent crime rates per 100,000 person years. When the temperature contemporaneous month was used in the analysis, the correlation coefficients were −0.46 (p = 0.13) with Bmax of controls, −0.68 (p = 0.02) with Bmax of violent offenders, and 0.75 (p = 0.005) with violent crime rate. The plots for those correlations are shown in Supplementary Figure S3.

The B_max_ values for the platelet 5-HT reuptake sites (SERT) are presented in Supplementary Table S1 and Supplementary Figure S2. The mean B_max_ values for SERT showed a clear seasonal fluctuation for both groups, with a maximum in February and minimum value in July. Figure 5 shows the correlations between ambient temperature, peripheral serotonin transporter densities and violent crime rates. Among healthy individuals, an analysis during a 12-month period showed that ambient temperature, with a one-month delay, correlated significantly with peripheral serotonin transporter density (r = −0.64, p = 0.0025, df = 12), which itself correlated negatively with the incidence of violent crime (r = −0.56, p = 0.06). While ambient temperature of the preceding month explained 34% of the variance in violent crime rate during the 12-month period, its contribution was diluted to 13% (partial correlation co-efficient 0.36) when SERT density was used as a co-variate. Among violent offenders, the correlation between SERT density and ambient temperature with a one-month delay was even stronger (r = −0.68, p = 0.02, df = 11), and SERT density explained 39% of the variance in the violent crime rate (r = −0.63, p = 0.039). When the SERT density of violent offenders was used as a co-variate, the contribution of ambient temperature of the preceding month to the incidence of violent crime decreased from 34% to 15% (partial correlation co-efficient 0.38). The results for contemporaneous month are shown in Supplementary Figure S3. In that analysis the correlation was weaker (r = −0.46, vs. −0.64) between B_max_ of controls and ambient temperature but slightly stronger between B_max_ of offenders and ambient temperature (r = −0.683 vs. −0.676) than for one-month delay analysis. Correlations between the monthly sum of sunshine hours (normalized with the number of days/month) vs. SERT densities (r = −0.15, p = 0.64 among healthy individuals; r = −0.35, p = 0.29 among violent offenders) were weaker than those for ambient temperature. The correlation between sunshine hours and montly violent crime rate was 0.60, p = 0.04.

## Discussion

These results demonstrate that low 5-HT transporter density and high ambient temperatures are associated with higher rates of violent crimes in Finland. Over a 16-year period, increases in ambient temperature accounted for 24% of the seasonal variation in violent crime without monthly stratification, and 10% when stratified by month. An additional analysis during a 12-month period showed that ambient temperature correlated with a one month delay in the seasonal changes in peripheral SERT density (r = −0.64 among healthy subjects and −0.68 among violent offenders), which itself correlated strongly with monthly violent crime rate (r from −0.56 to −0.63, respectively). This implies that the seasonal variation in violent offending is influenced by natural fluctuations in the serotonergic system, and that a 2 °C increase in ambient temperature could increase the violent crime rate by more than 3% if other contributing factors remained constant. This estimate is close to the results published by Ranson^10^, which suggest that climate change will increase the incidence of murder in the United States by 2.2%, and the incidence of aggravated assaults by 2.3%, by end of the century. Concerning absolute numbers of violent crime, Anderson *et al*. have estimated that 8 °F (corresponding to about 4 °C) warming will result to 100,000 additional murders/assults in US^26^. Two previous studies on this topic have suggested that the positive correlation between ambient temperature is non-linear, and that incidence of violent crime does not increase further when the ambient temperature is higher than 27–32 °C^8, 27^. However, these findinds may be explained by shortcomings in analytical methods, and the correlation is more linear when time of the day is taken into account in the statistical analysis^28^. Although it seems plausible that the correlation between ambient temperature and aggressive behaviour exists also beyond temperatures higher than 27 °C, our results can be generalized with reasonable quantitative certainty only to non-tropical and non-subtropical areas, where mean temperatures for all months are below 25 °C, with substantial seasonal variations in temperature.

Global warming may increase violent behavior by worsening of developmental environment of children, by causing war and civil unrest, and by direct effect on the human physiology^29^. Our finding of a substantial inverse correlation between monthly mean B_max_ and monthly assault occurrence rates is in line with reports of reduced platelet imipramine B_max_ among adolescents with conduct disorder^22^ and impulsive-aggressive children^21^, as well with decreased cerebral SERT densities among suicidal individuals^30, 31^. On the other hand, our violent offenders had higher B_max_ values than healthy individuals. A similar finding has been reported by Coccaro *et al*.^20^ who obtained higher SERT densities from patients with personality disorders and aggressive behaviour, when compared to healthy individuals. However, within the aggressive patient group the B_max_ values had an inverse correlation with aggressivity^20^. Because most all of our patients were habitual violent offenders, it was not possible to use standard aggression scales to analyze the correlation between B_max_ and aggression, as the majority of them would have had maximum scores. In conclusion, alterations in SERT levels differ between depressed suicidal patients, impulsive children and adult antisocial individuals, thus reflecting heterogeneous dysfunctions of serotonergic system. Our results indicate that the serotonergic system responds to increasing ambient temperature with a one month delay. It is plausible to suggest that the gradient in the seasonal change of SERT levels is a major factor related to aggression, while the absolute amount of SERT activity (as B_max_) does not play a major role. We presume that alterations in platelet SERT density reflect analogous alterations in the brain, since previous studies have shown that SERT densities in the brain are higher during the autumn and winter when compared to spring and summer, and that there is a negative correlation between these densities and the duration of daily sunshine^32^. Also, light therapy is known to decrease SERT density in the brain^33^. Although hours of sunshine had a somewhat similar correlation with peripheral SERT densities in our study, ambient temperature had substantially stronger correlations with both SERT densities and the occurrence of violent crime. Moreover, when the months were stratified, the correlation became weakly negative between sunshine and the violent crime rate. This suggests that ambient temperature may be the most important environmental factor that modifies aggressive behavior in humans. However, it must be acknowledged that we had data on SERT only for one year period, and it was not possible to stratify month in the one-year analysis to control the effect of other seasonal variables than temperature such as summer vacation. In the context of criminality, ambient temperature does not provide only the motive to commit a crime (increased impulsivity and irritability influenced by serotonergic system), but it does influence also opportunity. Put simply, violent crimes tend to be committed during nice weather (i.e., summer vs. winter).

To our knowledge, this is the first report to demonstrate that a seasonal variation in violent crime is associated with weather conditions and 5-HT function. However, this association does not necessarily indicate causation. In order to address the question of causation, we must consider the relationships that concern the associations between ambient temperature, serotonin transporter density and the occurrence of violent crime. To this end, we stratified the months to eliminate the effect of factors other than weather, such as the holiday season (i.e., we compared the results for cold January vs. mild January, or hot July with chilly July, and so on). By logic, it is extremely unlikely that a) SERT density or violent crime rate could affect ambient temperature, b) violent crime rates could affect SERT densities, or c) there could be a common causal factor other than the seasonal variation of solar radiation that could influence ambient temperature, SERT density, and violent crime. Therefore, it seems to be obvious that ambient temperature drives variations in SERT levels and the occurrence of violent crime. Either high ambient temperature and/or related sunshine has a solely direct effect on brain serotonergic activity, or the effect is mediated partly through intermediating factors. The analysis of 18-year period showed that although there was a substantial correlation between the mean ambient temperature of the preceding month versus the monthly violent crime rate (r = 0.40), agreeing with the assumption that ambient temperature modifies SERT-levels and violent behavior with one-month delay. However, the correlation was even stronger between mean ambient temperature of the current month versus the violent crime rate of the same month (r = 0.51). This indicates that the association between ambient temperature and violent behavior is multifactorial. Since a large body of literature implies that serotoninergic activity is strongly related to impulsive and aggressive behavior^16, 17, 20, 34^, it is reasonable to suggest that changes in serotonin transporter density modify the incidence of violent behavior, and that seasonal effects of solar radiation influence both. Recent studies indicate that, in animals, whole-body heating activates subdivisions of the dorsal raphe nucleus implicated in mood regulation, and whole-body warming has a substantial antidepressant effect in humans^35^. It is plausible that when temperature rises, people go out more and meet other people, which increases the rate of violent incidents. Therefore, the increase of violent crime rate is attributable also to serotonergic activation of the victims, and not only that of offenders. From an evolutionary point of view, ambient weather conditions have a direct impact on an eternal question for our species; should I stay or should I go? In particular, seasonal “bad” weather conditions (i.e., low ambient temperature, low amounts of sunshine) up-regulate 5-HT function, thereby decreasing impulsivity and risk-taking; e.g., influencing one to not wander too far from the homesite in bad weather. In contrast, “fine” weather (i.e., high ambient temperature and sunshine) may down-regulate 5-HT function and contribute to higher impulsivity and novelty seeking, such as hunting and other forms of useful aggression.

## Materials and Methods

Monthly violent crime rates (total N = 551,529 during the years of 1996–2013) were obtained from the Statistics Centre of Finland. Violent crime was defined as homicide, attempted homicide, aggravated assault, attempted aggravated assault, assault and attempted assault. These rates (monthly number of crimes divided by the number of days in the month) were correlated (random-effects Poisson regression stratified by month) with the monthly mean temperatures from January 1996 to December 2013, obtained from the Finnish Meteorological Institute’s measurement station in Jokioinen, 60°49′N and 23°30′E, which is near the geographic center of the Finnish population. The monthly mean B_max_ values were collected from volunteers in Kuopio from December 1996 – November 1997 and correlated (Pearson correlation) with the meteorological values obtained during the same 12-month period from the Finnish Meteorological Institute’s Kuopio measurement station (63°01′N and 27°48′E). Also, correlations between SERT B_max_ values and monthly nationwide violent crime values (total N = 25,084 during 12 months) were calculated.

Monthly blood samples were analyzed from 33 male violent offenders (aged 34.5 ± 9.8 years, from December 1996 to November 1997) who were committed to a forensic mental examination at the Niuvanniemi high security state mental hospital in Kuopio, Finland, and 18 healthy males aged 31.4 ± 5.0 years (mean ± S.D.), who received no medication and lived within 50 km from the town of Kuopio (63°01′N and 27°48′E). The present study used data gathered form the previously published study by Callaway *et al*.^14^. All methods were carried out in accordance with relevant guidelines and regulations. Permission for that study and all experimental protocols used were granted by the Medical Director of Niuvanniemi Forensic Psychiatric Hospital, and all participants gave their voluntary informed consent. (The formal ethical committee procedure was established in 1999 in Finland.)

The average length of the hospital stay for the violent offenders, prior to sampling, was 9.7 days (SD ± 10.5, range 0–40 days), with no access to alcohol or other unprescribed drugs during their incarceration. Five of the 38 screened offenders were prescribed an antidepressant, and these individuals were excluded from the final analysis. Nineteen (58%) of the remaining 33 offenders had no prescribed medication at the time of blood sampling. Fourteen offenders (42%) were prescribed various medications, with some overlap, which included ibuprofen (2), ketoprofen (1), enalapril (ACE-inhibitor) (1), beta-blockers; sotalol (1) and atenolol (1), miconazole (1), glucocorticoid (1), zopiclone (5), lanzoprazol (1), nitzatidin (1), hydroxidizine (4), clonazapam (2), biperidine (2) and chlordiazepoxide (1). The majority of offenders had recently been convicted of a violent crime such as murder, manslaughter, rape, kidnapping, armed robbery, assault, arson, in addition to the recurrent use/misuse of alcohol, and five of the violent offenders (15%) were without a prior conviction for a previous violent crime. None of the violent offenders suffered from a DSM axis-1 mental disorder, aside from substance abuse.

### Statistical calculations

The incidence of monthly violent crimes per 100,000 person years were normalized by taking into account the number of days in each month. The incidence and rate ratios (IRR) were estimated by using the Random-effects Poisson regression models with 95% confidence intervals (CIs), stratified by month. Nonlinear associations between temperature and violent crimes were modelled by using restricted cubic splines. The analyses were performed by Stata/SE version 10.1 for Windows (StataCorp. LP).

### Blood sampling: collection of PRP from plasma

Platelet rich plasma (PRP) samples were collect in the morning between 7:00–9:00 throughout the study period. Briefly, whole blood was collected in glass tubes that contained 0.13% EDTA (13 mg/10 ml tube) as an anticoagulant. These samples were centrifuged at 250 *g* for 10 min, and the PRP (supernatant) was immediately transferred to clean plastic tubes for storage at −30 °C prior to analysis. At least 10 ml of PRP was required for the complete binding analysis of each sample. Concentrated homogenates were prepared as previously described^36, 37^.

### Protein assay

Protein concentrations of platelet homogenates were assayed using a Coomassie protein assay kit from Bio-Rad (Bradford, 1976), using bovine serum albumin (Sigma) as a protein standard, prior to SERT B_max_ analyses.

### [3H]Citalopram binding assay

Eight concentrations of [^3^H]citalopram (specific activity 85.5 Ci/mmol, Du Pont NEN, Boston, MA), ranging from 0.05 to 6.00 nmol were used to determine *K*_d_ in solutions of isolated platelets. B_max_ was calculated from an additional single point analysis at 5.5 nmol in triplicate (i.e. non-specific binding plus the sample in duplicate). Specific binding was defined as the total binding minus binding in the presence of 0.10 μmol paroxetine (Beecham Pharmaceuticals, Gf. Burgh, Epsom, Surrey, UK). Incubation was at room temperature for 1.5 h. Membranes were recovered and washed with ice cold incubation buffer through Whatman *GF/B* filters using a 96 channel harvester (Brandel 96, Gaithersburg, MD), where filter-bound [3H]citalopram did not exceed 5%. Radioactivity was calculated by a Wallach 1450 MicroBeta Trilux liquid scintillation counter, as previously described^36^.

## Electronic supplementary material

Supplementary Information
