# Supplementary material for: The Association of Ambient Temperature and Violent Crime
Source: Sci Rep. 2017 Jul 28;7:6543. doi: 10.1038/s41598-017-06720-z (PMC5533778; doi:10.1038/s41598-017-06720-z)
Supplement: Supplementary file 1 — Supplementary Information [file 41598_2017_6720_MOESM1_ESM.pdf]

Supplementary Information

The Association of Ambient Temperature and Violent Crime

Jari Tiihonen, Pirjo Halonen, Laura Tiihonen, Hannu Kautiainen, Markus Storvik, James Callaway

Month	Healthy individuals			Male violent offenders		
	B_{max}	S.D.	N	B_{max}	S.D.	N
Jan 1997	1486	472	18	1832	447	6
Feb 1997	1703	577	18	2133	430	3
Mar 1997	1406	442	18	1620	562	5
Apr 1997	1396	587	18	1588	228	5
May 1997	1456	649	18	2028	–	1
Jun 1997	1563	702	18	1415	139	2
Jul 1997	1157	366	17	1403	542	3
Aug 1997	1347	413	17	896	682	2
Sep 1997	1355	387	17	1690	74	2
Oct 1997	1355	438	16	–	–	0
Nov 1997	1236	380	17	1706	609	2
Dec 1996	1447	617	18	1802	639	2

Supplementary Table S1. Complete data set for the averaged monthly B_{max} (in fmol/mg protein) and SEM from human blood platelets over one year. The set consisted of 18 males who were followed up for 12 months, and 33 violent offenders, each studied once. (The follow-up started in December 1996 and ended in November 1997.)

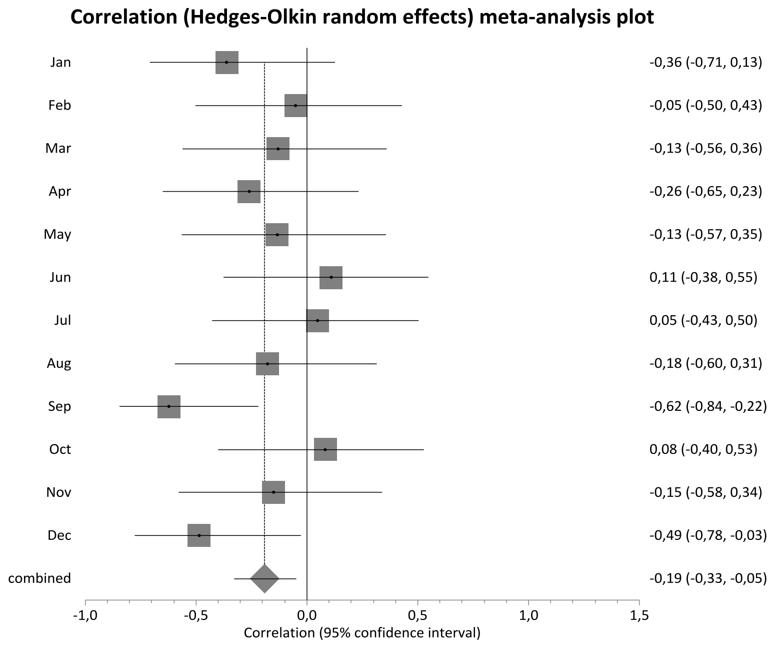

Supplementary Figure S1. Monthly sum of direct sunshine hours (normalized with the number of days/month) and monthly violent crime rate during 18-year follow-up (N = 18, r = -0.19, p = 0.04).

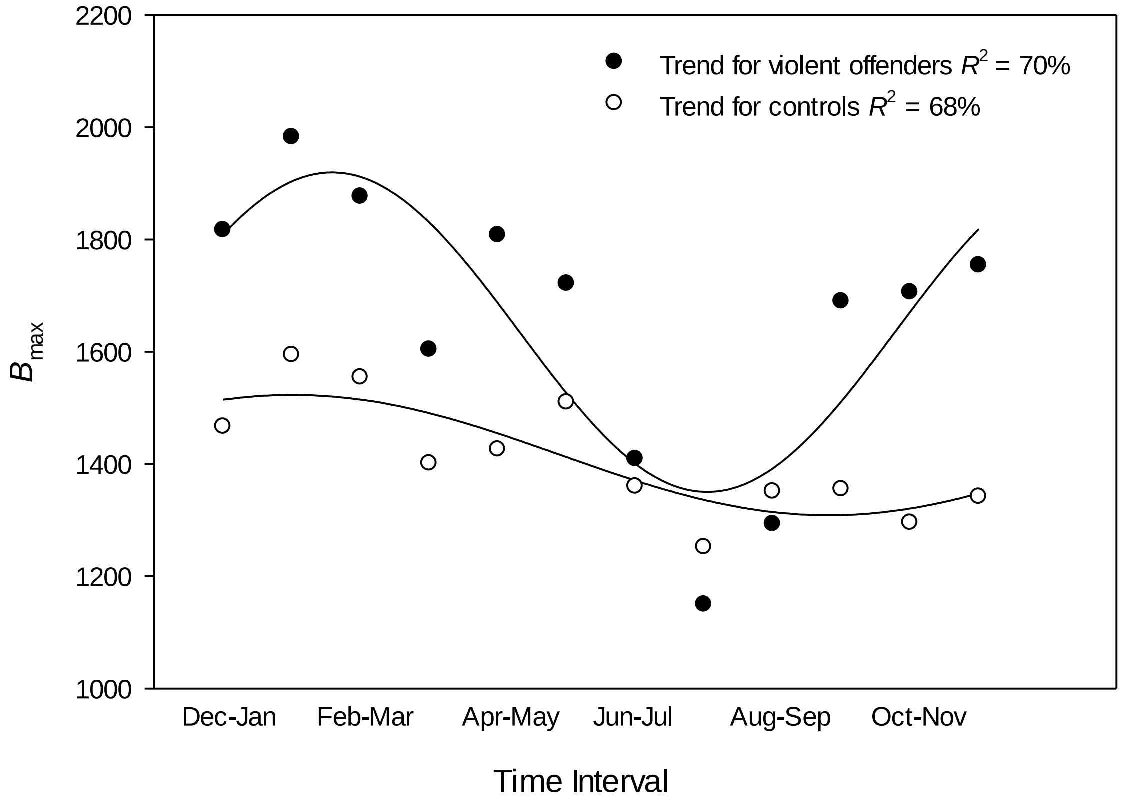

Supplementary Figure S2. The seasonal variation of peripheral transporter density (B_{max}) among healthy individuals (N = 18; each individual studied monthly) and violent offenders (N = 33; each individual studied only once). (The follow-up started in December 1996 and ended in November 1997.) The highest B_{max} values were observed during winter and the lowest values during summer, indicating seasonal variation by monthly values [(R^2 = 68%, F=15.8, p = 0.00011, df = (1, 16), repeated measures ANOVA for healthy individuals; R^2 = 70%, F=7.1, p = 0.021, df = (1, 12) oneway ANOVA for violent offenders)].

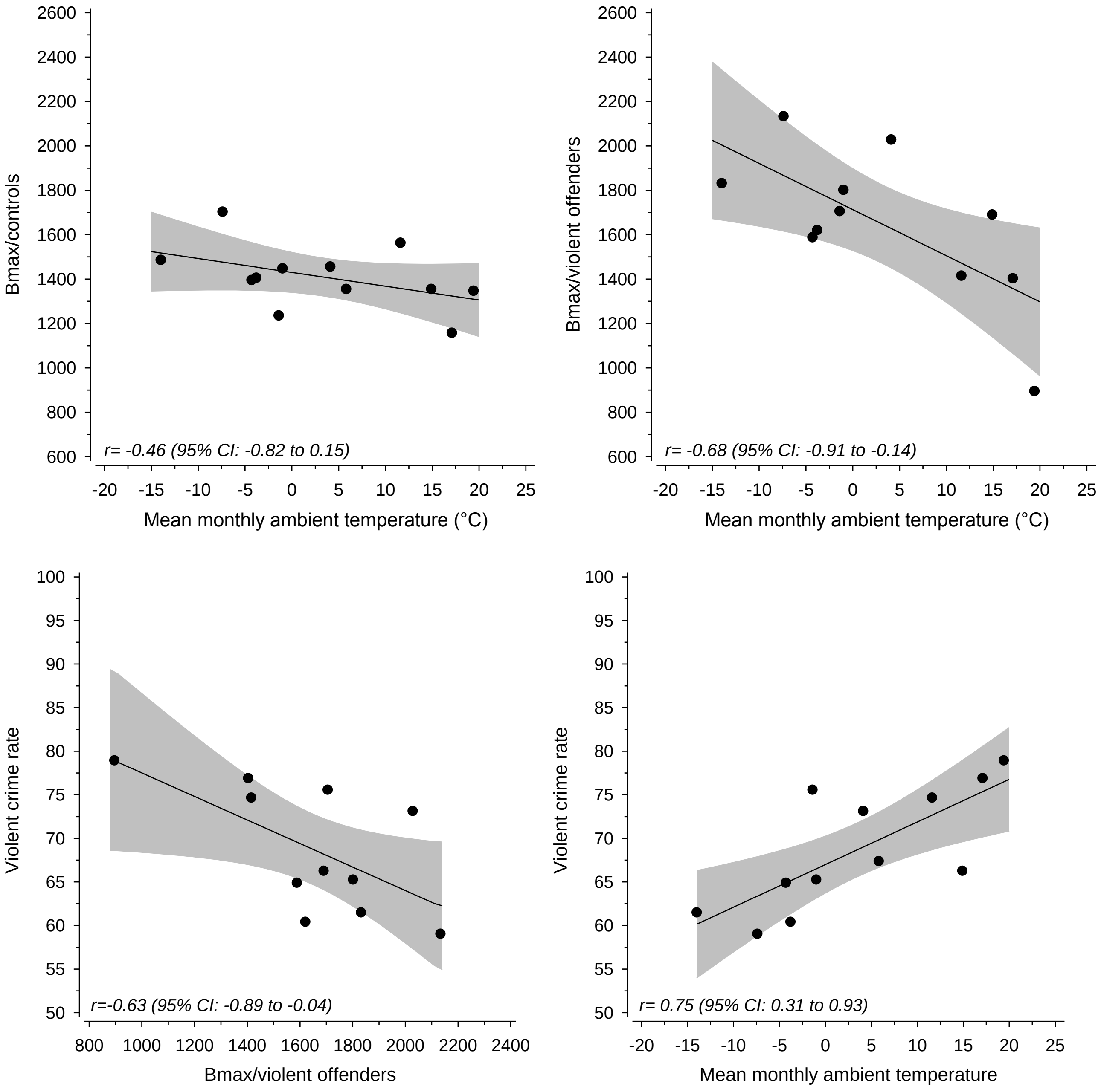

Supplementary Figure S3. The correlations between ambient temperature of contemporaneous month, peripheral SERT denties (B_{max}, as fM of ligand/mg of protein) and violent crime rates per 100,000 person years.
